# The effectiveness of mindfulness-based stress reduction intervention on alleviating anxiety and depression in postoperative patients with cervical cancer: A protocol for systematic review and meta-analysis

**DOI:** 10.1097/MD.0000000000028706

**Published:** 2022-02-04

**Authors:** Xiaoju Yang, Li Huang, Chunlin Li, Ning Ji, Hongcheng Zhu

**Affiliations:** a Department of Obstetrics and Gynaecology, The Central Hospital Of Enshi Tujia And Miao Autonomous Prefecture, Enshi, Hubei Province, China; b Department of Infectious Disease, Enshi Tujia & Miao Autonomous Prefecture Center for Disease Control and Prevention, Enshi, Hubei Province, China; c Radiology Center, The Central Hospital Of Enshi Tujia And Miao Autonomous Prefecture, Enshi, Hubei Province, China; d Department of Obstetrics and Gynaecology, Hubei Xianfeng County Hospital of Traditional Chinese Medicine, Enshi, Hubei Province, China.

**Keywords:** anxiety, cervical cancer, depression, meta-analysis, mindfulness-based stress reduction, protocol

## Abstract

**Background:**

Surgical treatment for cervical cancer, as a stressor, largely leads to strong psychological reactions to stress like anxiety and depression. Whether mindfulness-based stress reduction (MBSR) can alleviate anxiety and depression in patients after cervical cancer surgery is controversial. Therefore, we aim to perform a meta-analysis involving randomized controlled trials analyzing the effect of MBSR on alleviating anxiety and depression in patients after cervical cancer surgery, thus providing evidence-based medical evidences for nonpharmacological interventions.

**Methods:**

Randomized controlled trials analyzing the effect of MBSR on alleviating anxiety and depression in patients after cervical cancer surgery will be searched in online databases, including Cochrane Central Register of Controlled Trials Repositories, PubMed, Embase, Web of Science, Chinese Science Citation Database, China National Knowledge Infrastructure, Chinese Biomedical Literature Database, Chinese Scientific Journal Database, and Wan Fang Data. After screening eligible studies, we will perform a meta-analysis on the effect of MBSR on alleviating anxiety and depression in patients after cervical cancer surgery.

**Results:**

The results of this meta-analysis will be submitted to a peer-reviewed journal for publication.

**Conclusion:**

This study will provide reliable evidence-based evidences for the effects of MBSR on alleviating anxiety and depression in patients after cervical cancer surgery.

**Ethics and dissemination:**

Ethical approval was not required for this study. The systematic review will be published in a peer-reviewed journal, presented at conferences, and shared on social media platforms.

**OSF Registration number:**

DOI 10.17605/OSF.IO/EXUM3.

## 1 Introduction

Cervical cancer is one of the most common gynecological malignancies. It is reported that about 200,000 women die of cervical cancer each year worldwide, and more than 130,000 new cases of cervical cancer are diagnosed each year in China.^[1–3]^ Cervical cancer is usually treated by surgery after diagnosis. As a kind of stressor, surgical treatment of cervical cancer will, to a large extent, lead to strong stress psychological reactions like anxiety and depression. Improper handling of stress response can cause psychological disorders to varying extents.^[4,5]^ Adverse psychological conditions like anxiety, depression and fear can negatively affect psychological and emotional well-being and reduce the quality of survival of patients.^[6,7]^ Therefore, interventions to alleviate negative emotions in patients after cervical cancer surgery are particularly important.

Mindfulness-based stress reduction (MBSR) is a nonpharmacological intervention based on positive thinking, which enhances the ability to live with stress and improves quality of life by positive thinking sessions involving positive diet, meditation, body scanning, yoga, nonjudgmental attitudes, and management of stressors and emotions.^[8–10]^ It has been successfully applied in foreign countries for emotion management, stress relief and clinical treatment of illnesses.^[11,12]^ At present, MBSR is widely used in the field of psychotherapy at abroad. Moreover, it has been used in the treatment of cervical cancer patients in other countries, which has achieved positive results.^[13]^

MBSR has become one of the treatment methods for the systematic treatment of cancer patients at home and abroad. MBSR can be used as a nonpharmacological intervention for postoperative psychological intervention in cervical cancer. However, clinical evidences are scant.^[14,15]^ Therefore, in this study, we perform a meta-analysis to assess the effect of MBSR on alleviating anxiety and depression levels in patients after cervical cancer surgery.

## 2 Methods

### 2.1 Protocol

Under the guidance of the preferred reporting items for systematic reviews and meta-analysis protocols, this protocol of systematic review and meta-analysis has been drafted.^[16]^ The research framework has been registered on the open science framework (Registration Number: DOI 10.17605/OSF.IO/EXUM3).

### 2.2 Ethics

Since this is a protocol without patient recruitment and personal information collection, the approval of the ethics committee is not required.

### 2.3 Eligibility criteria

#### 2.3.1 Types of studies

i) Randomized controlled trials;
ii) Publication language in Chinese or English;
iii) Outcome indicators will include testing data on depression and anxiety.

#### 2.3.2 Types of participants

Adult patients over 18 years old after cervical cancer surgery.

#### 2.3.3 Types of interventions

Patients in the control group will be given conventional care measures, while those in the experimental group will receive MBSR.

#### 2.3.4 Types of outcome measurements

Any rating scale that describes anxiety and depression.

### 2.4 Exclusion criteria

1. Studies with incomplete data;
2. Repeatedly published literatures;
3. Review articles, techniques, case reports, letters to the editor, and editorials.

### 2.5 Searching strategy

We will systematically search relevant randomized controlled trials published before January 2022 in the following databases: Cochrane Central Register of Controlled Trials Repositories, PubMed, Embase, Web of Science, Chinese Science Citation Database, China National Knowledge Infrastructure, Chinese Biomedical Literature Database, Chinese Scientific Journal Database, and Wan Fang. Searching strategy in Pubmed was shown in Table 1, which will be suitable in searching other online databases.

**Table 1:** PubMed search strategy.

Number	Search terms
#1	Uterine cervical neoplasms[MeSH]
#2	Cancer of cervix[Title/Abstract]
#3	Cancer of the cervix[Title/Abstract]
#4	Cancer of the uterine Cervix[Title/Abstract]
#5	Cervical cancer[Title/Abstract]
#6	Cervical neoplasms[Title/Abstract]
#7	Cervix cancer[Title/Abstract]
#8	Cervix neoplasms[Title/Abstract]
#9	Neoplasms, cervical[Title/Abstract]
#10	Neoplasms, cervix[Title/Abstract]
#11	Uterine cervical cancer[Title/Abstract]
#12	Cancer, cervix[Title/Abstract]
#13	Cancer, uterine cervical[Title/Abstract]
#14	Cancers, cervix[Title/Abstract]
#15	Cancers, uterine cervical[Title/Abstract]
#16	Cervical cancer, uterine[Title/Abstract]
#17	Cervical cancers, uterine[Title/Abstract]
#18	Cervical neoplasm[Title/Abstract]
#19	Cervical neoplasm, uterine[Title/Abstract]
#20	Cervical neoplasms, uterine[Title/Abstract]
#21	Cervix neoplasm[Title/Abstract]
#22	Neoplasm, cervical[Title/Abstract]
#23	Neoplasm, cervix[Title/Abstract]
#24	Neoplasm, uterine cervical[Title/Abstract]
#25	Neoplasms, Uterine cervical[Title/Abstract]
#26	Uterine cervical cancers[Title/Abstract]
#27	Uterine cervical neoplasm[Title/Abstract]
#28	OR/1–27
#29	Mindfulness-based stress reduction[Title/Abstract]
#30	MBSR[Title/Abstract]
#31	OR/29–30
#32	Randomized controlled trials as topic[MeSH]
#33	Clinical trials, randomized[Title/Abstract]
#34	Controlled clinical trials, randomized[Title/Abstract]
#35	Trials, randomized clinical[Title/Abstract]
#36	Random∗[Title/Abstract]
#37	OR/32–36
#38	#28 and #31 and #37

### 2.6 Data screening and extraction

**Figure 1:**
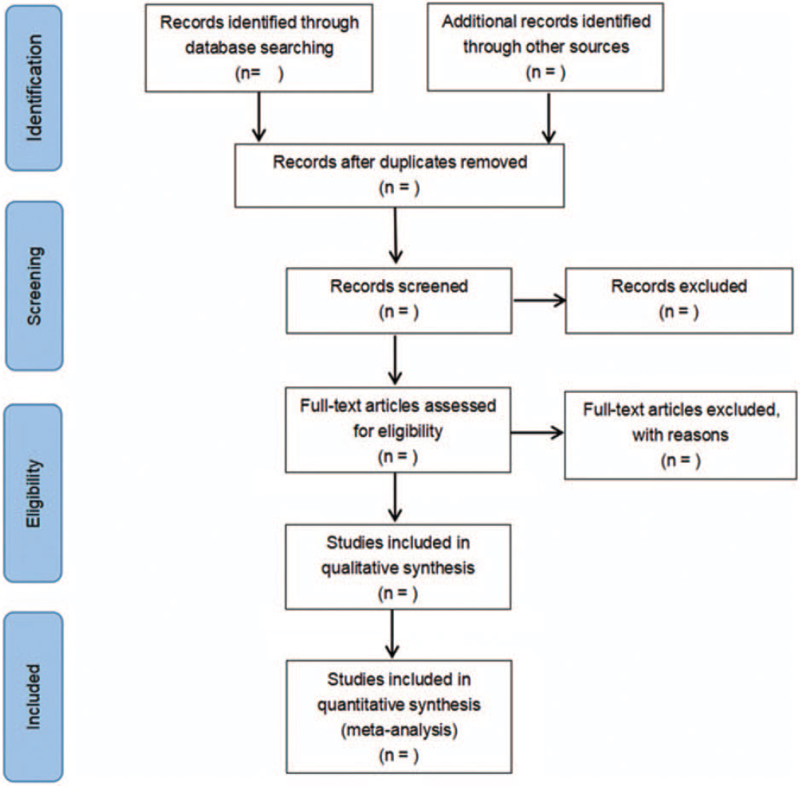
Flow diagram of literature retrieval.

The literature selection process was listed in Figure 1. Two investigators will independently review all abstracts and full-texts according to inclusion and exclusion criteria. Any disagreement will be resolved through a discussion with a third investigator.

Two investigators will extract data from eligible studies using a predesigned information sheet and cross-check them. Any uncertainty will be solved by discussing with the third investigator. The following data will be extracted: first, author, year of publication, study population, study type, interventions, time of measurement, and relevant outcome indicators.

### 2.7 Quality evaluation

Two authors will independently assess the risk of bias of included studies using Cochrane Collaboration risk of bias assessment tool, and all disagreements will be resolved by discussing with a third investigator.

### 2.8 Statistical analysis

Rev-Man 5.3 software will be applied for the meta-analysis. The pooled effects will be estimated by using the standardized mean differences and its 95% confidence interval. Heterogeneity between studies will be assessed by I-square (*I*^*2*^) and Q-statistic (*P* < .10), and *I*^*2*^ > 50% will be recognized as heterogeneity.^[17]^ If *P* ≥ .1 and *I*^*2*^ ≤ 50%, a fixed-effect model (Mantel–Haenszel method) will be adopted for analysis: otherwise, a random-effect model will be used.

#### 2.8.1 Dealing with missing data

Insufficient or missing data in the literature will be obtained by e-mailing the authors. If data are still not available, only the current available data will be analyzed and the potential impacts will be discussed.

#### 2.8.2 Subgroup analysis

Subgroup analysis will be carried out according to the duration of intervention.

#### 2.8.3 Sensitivity analysis

We will conduct sensitivity analysis by analyzing the remaining studies after removing one study at each time.

#### 2.8.4 Publication bias

If the number of included studies is no less than 10, a funnel chart will be used to assess publication bias.^[18,19]^

## 3 Discussion

Recently, various nonpharmacological interventions on alleviating negative emotions after cancer surgery have emerged and increasingly applied.^[20–22]^ There is growing evidence that MBSR can alleviate anxious and depressive symptoms, and promote healthy outcomes in patients after cervical cancer surgery. MBSR is a cognitive therapy based on mindfulness meditation aiming to cultivate positive thinking. It helps people to develop the ability to cope with the present experience in a nonjudgmental or open-hearted way.^[23,24]^ MBSR has been widely used clinically, commonly in breast cancer patients to reduce psychological symptoms and enhance positive psychological constructs, which has obtained a significant success.^[25–27]^ However, the specific effects of MBSR on alleviating anxiety and depression in patients after cervical cancer surgery may differ from the subject, timing, frequency, method, and duration of the intervention. Based on the principles and methods of evidence-based medicine, the present study aims to further clarify the effects of MBSR on alleviating anxiety and depression in patients after cervical cancer surgery, and to provide a basis for clinical application.

## Author contributions

**Conceptualization:** Hongcheng Zhu, Xiaoju Yang.

**Data curation:** Li Huang.

**Formal analysis:** Li Huang.

**Funding acquisition:** Hongcheng Zhu.

**Investigation:** Li Huang.

**Methodology:** Li Huang, Chunlin Li.

**Project administration:** Hongcheng Zhu.

**Resources:** Chunlin Li, Ning Ji.

**Software:** Chunlin Li, Ning Ji.

**Supervision:** Hongcheng Zhu.

**Validation:** Ning Ji.

**Visualization:** Ning Ji.

**Writing – original draft:** Hongcheng Zhu, Xiaoju Yang.

**Writing – review & editing:** Hongcheng Zhu, Xiaoju Yang.

## References

[1] Swaine JG Parish SL Luken K. Breast and cervical cancer screening for women with intellectual disabilities. Health Soc Work 2013;38:183–6. 24437024 10.1093/hsw/hlt012

[2] Shrestha AD Neupane D Vedsted P Kallestrup P. Cervical cancer prevalence, incidence and mortality in low and middle income countries: a systematic review. Asian Pac J Cancer Prev 2018;19:319–24. 29479954 10.22034/APJCP.2018.19.2.319 PMC5980914

[3] Yin N Chen J Li Y. Influence of psychological intervention on anxiety and depressive symptoms in patients with cervical cancer: a meta-analysis. Chin Nurs Res 2017;31:799–801.

[4] Mamguem Kamga A Dumas A Joly F. Long-term gynecological cancer survivors in Côte d’Or: health-related quality of life and living conditions. Oncologist 2019;24:e490–500. 30578310 10.1634/theoncologist.2018-0347 PMC6656453

[5] Fu T Guang HJ Gao XZ. Percutaneous nerve electrical stimulation for fatigue caused by chemotherapy for cervical cancer. Medicine (Baltimore) 2018;97:e12020. 30313023 10.1097/MD.0000000000012020 PMC6203554

[6] Lu D Andrae B Valdimarsdóttir U. Psychological distress is associated with cancer-specific mortality among patients with cervical cancer. Cancer Res 2019;79:3965–72. 31253667 10.1158/0008-5472.CAN-19-0116

[7] Printz C. Psychological stress is associated with a higher risk of cervical cancer mortality. Cancer 2020;126:240–1. 31917471 10.1002/cncr.32686

[8] Yan L Zhang Q Zhu B Li J. Research progress of mindfulness-based stress reduction for symptom intervention in cancer patients. J Nurses Train 2019;34:39–43.

[9] Robb SW Benson K Middleton L Meyers C Hébert JR. Mindfulness-based stress reduction teachers, practice characteristics, cancer incidence, and health: a nationwide ecological description. BMC Complement Altern Med 2015;15:24. 25887555 10.1186/s12906-015-0545-3 PMC4342874

[10] Adelian H Khodabandeh Shahraki S Miri S Farokhzadian J. The effect of mindfulness-based stress reduction on resilience of vulnerable women at drop-in centers in the southeast of Iran. BMC Womens Health 2021;21:255. 34167523 10.1186/s12905-021-01390-6 PMC8222952

[11] Schellekens MPJ Jansen ETM Willemse HHMA van Laarhoven HWM Prins JB Speckens AEM. A qualitative study on mindfulness-based stress reduction for breast cancer patients: how women experience participating with fellow patients. Support Care Cancer 2016;24:1813–20. 26446701 10.1007/s00520-015-2954-8 PMC4766203

[12] Würtzen H Dalton SO Christensen J. Effect of mindfulness-based stress reduction on somatic symptoms, distress, mindfulness and spiritual wellbeing in women with breast cancer: results of a randomized controlled trial. Acta Oncol (Stockholm, Sweden) 2015;54:712–9. 10.3109/0284186X.2014.997371 25752972

[13] Zhang H Li Y Li M Chen X. A randomized controlled trial of mindfulness-based stress reduction for insomnia secondary to cervical cancer: sleep effects. Appl Nurs Res 2019;48:52–7. 31266608 10.1016/j.apnr.2019.05.016

[14] Zang J Li T Zhang M Ji A. The effect of mindfulness decompression therapy on anxiety and depression level of postoperative patients with cervical cancer. Psychol Mon 2021;16:68–9.

[15] Ren C. Effect of cognitive-behavioral combined with mindfulness-based stress reduction on posttraumatic growth in young postoperative cervical cancer patients. Today Nurse 2020;027:98–101.

[16] Shamseer L Moher D Clarke M. Preferred reporting items for systematic review and meta-analysis protocols (PRISMA-P) 2015: elaboration and explanation. BMJ (Clin Res ed) 2015;350:g7647. 10.1136/bmj.g7647 25555855

[17] Higgins JP Thompson SG Deeks JJ Altman DG. Measuring inconsistency in meta-analyses. BMJ (Clin Res ed) 2003;327:557–60. 10.1136/bmj.327.7414.557 PMC192859 12958120

[18] Irwig L Macaskill P Berry G Glasziou P. Bias in meta-analysis detected by a simple, graphical test. Graphical test is itself biased. BMJ (Clin Res ed) 1998;316:470 author reply 470–71. PMC2665595 9492687

[19] Yang Y Ma QY Yang Y. Evidence-based practice guideline of Chinese herbal medicine for primary open-angle glaucoma (qingfeng-neizhang). Medicine (Baltimore) 2018;97:e0126. 29595636 10.1097/MD.0000000000010126 PMC5895393

[20] Shi X Ma L Hao J Yan W. Regulatory effects of comprehensive psychological intervention on adverse emotions and immune status of cervical cancer patients during the perioperative period. Am J Transl Res 2021;13:6362–71. 34306375 PMC8290739

[21] Wenzel L Osann K Hsieh S Tucker JA Monk BJ Nelson EL. Psychosocial telephone counseling for survivors of cervical cancer: results of a randomized biobehavioral trial. J Clin Oncol 2015;33:1171–9. 25713429 10.1200/JCO.2014.57.4079 PMC4372853

[22] Han D Wang D Yang J Li X. Effect of multidisciplinary collaborative continuous nursing on the psychological state and quality of life of patients with cervical cancer. Am J Transl Res 2021;13:6654–61. 34306409 PMC8290774

[23] Baer RA. Using self-report assessment methods to explore facets of mindfulness. Assessment 2006;13:27–45. 16443717 10.1177/1073191105283504

[24] Nejad FK Shahraki KA Nejad PS Moghaddam NK Jahani Y Divsalar P. The influence of mindfulness-based stress reduction (MBSR) on stress, anxiety and depression due to unwanted pregnancy: a randomized clinical trial. J Prev Med Hyg 2021;62:E82–e88. 34322621 10.15167/2421-4248/jpmh2021.62.1.1691 PMC8283654

[25] Haller H Winkler MM Klose P Dobos G Kümmel S Cramer H. Mindfulness-based interventions for women with breast cancer: an updated systematic review and meta-analysis. Acta Oncol (Stockholm, Sweden) 2017;56:1665–76. 10.1080/0284186X.2017.1342862 28686520

[26] Zhang Q Zhao H Zheng Y. Effectiveness of mindfulness-based stress reduction (MBSR) on symptom variables and health-related quality of life in breast cancer patients-a systematic review and meta-analysis. Support Care Cancer 2019;27:771–81. 30488223 10.1007/s00520-018-4570-x

[27] Zhang J Xu R Wang B Wang J. Effects of mindfulness-based therapy for patients with breast cancer: a systematic review and meta-analysis. Complement Ther Med 2016;26:01–10. 10.1016/j.ctim.2016.02.012 27261975

